# The Sore Head

**Published:** 1916-08

**Authors:** 


### The Sore Head.

It is plain to anyone interested in the economic and social problems of medicine, that what is popularly termed soreheadedness is a considerable and serious obstacle to professional unity, even to professional efficiency in its potential benefits to the community. If the sore head could be eliminated, the profession and the laity would both be immeasurably better off. But, it is only fair to recognize that the sore head is, to some degree, a secondary evil. That is to say, his Ishmaelism is more or less justified by the existence of primary evils to which his own attitude is merely a natural reaction.

Once we commented on the difference between two brothers, one of whom had risen far beyond his original status but whose success was checked by his general reputation of being tricky and dishonest, while the other, though in humble circumstances, seemed to be perfectly upright. "Bless you," was the reply, "—— (the second brother) is eating his heart out because he does not know enough to be as much of a shyster as —— (the former)." This is the trouble with many sore heads. It is not so much the existence of an ethical evil that troubles them as the fact that they are the victims and not the perpetrators of it.

We have heard of a physician, by no means a sore head, who, toward the close of an active independent career, very successful too, expressed the regret that, instead of becoming known as one who would always protest fearlessly against an evil, however prominent its source, he had not acquiesced and thus gained friendship and prestige from the very sources that he had antagonized. Such regret is itself regretable. The sore head is not only a general nuisance but he damages his own cause most of all. The essential trouble with him is his personal bias. He is thinking too much of the effect of general conditions on himself, too much of the individual source from which such conditions emanate. If he will think in terms of all who are injured; if he will act without malice to any one but against wrong conditions in general, he will cease to be a sore head and will exert a real influence toward reform.

